# Supplementary material for: Geometry image super-resolution with AnisoCBConvNet architecture for efficient cloth modeling
Source: PLoS One. 2022 Aug 24;17(8):e0272433. doi: 10.1371/journal.pone.0272433 (PMC9401171; doi:10.1371/journal.pone.0272433)
Supplement: Attachment — Submitted filename: Response_to_Reviewers.docx [file pone.0272433.s002.docx]

**Reviewer#1, Concern #1:** Why authors are used VGG19, because already best models are available like Xception Network, MobileNetwork etc.

**Author response:** Thank you for the good question. Because VGG19 network is used in a variety of applications using ConvNet, it has also been adopted in this paper. Our solution is not network-specific, and because it is stable in VGG19, it may be used and expanded to other ConvNet-based approaches. We will attempt to improve the algorithm in the future by applying it to the model you mentioned, the Xception Network.

**Author action:** We added the manuscript by “Line 444 in Section *Conclusion and Future Work*”.

**Reviewer#1, Concern #2:** If VGG19 response is better, plz shows the comparable results with Xception and Inception model.

**Author response:** In conclusion, we believe that employing Xception will provide results comparable to those obtained with VGG19. During the initial design phase of the algorithm, we thought about which one to choose between GAN and ConvNet. Due to the convolution filter, the detail of the feature map created by ConvNet is rather smoothed in comparison to GAN. However, because each pixel corresponds to a single vertex location in our method, the ConvNet with smoothed style was preferable to the GAN, which gives a detailed feature map via a nonlinear filter. As a result, we believe it will perform well in Xception, a ConvNet with 71 layers.

**Author action:** We added the manuscript by “Line 418 in Section *Results and Discussion*”.

**Reviewer#1, Concern #3:** Plz add latest reference 2020 and 2021 in your article.

**Author response:** Thank you for the good question.

**Author action:** We added the manuscript by in Section *Related Work*.

**Reviewer#1, Concern #4:** Why used the bicubic interpolation in Figure 7 to upscale the LR image to HR image, because interpolation is not designed for this purposes, all cuurent research replaced interpolation with deconvolution layer or subpixel layer to upscale the LR image to HR image.

**Author response:** The bicubic interpolation process can actually be omitted. In order to perform Image SR, the data that is resized by interpolating LR is learned through ConvNet, but there are also quite a few approaches that do not use interpolation. Since there are a lot of networks in the field of Image SR research, interpolation is an optional process that users can easily choose. As a result, we created the SR image by interpolating the LR image to match the size of the input data, and we employed bicubic interpolation rather than linear interpolation to achieve the better results.

**Author action:** Thanks for the good question.

**Reviewer#1, Concern #5:** if authors also calculated results on 3x and 4x scale it is better.

**Author response:** Similarly to how increasing the upsampling resolution does not always improve the results of image SR, our technique has limitations. While the nonlinear filter improves the quality of image and geometry SR, noise is frequently introduced by over-SR when the resolution is set too high. In our method, increasing the resolution by more than 3 times occasionally resulted in awkward upsampling results, and over-SR prevented wrinkle enhancement from performing properly. Experiments in a variety of scenes produced the most stable and satisfactory results at twice the resolution.

**Author action:** We added the manuscript by “Line 425 in Section *Results and Discussion*”.

**Reviewer#2, Concern #1:** After reading the section of related work, this section is detailed and comprehensive, but there are still some references that are closely related to this submission, they should be discussed carefully. I list them as following:

- Meng Zhang, Duygu Ceylan, Tuanfeng Wang, Niloy J. Mitra, Dynamic Neural Garments, arXiv 2021

- Tuanfeng Y. Wang, Tianjia Shao, Kai Fu, Niloy J. Mitra, Learning an intrinsic garment space for interactive authoring of garment animation, ACM Transactions on Graphics (TOG), vol. 38, no. 6, p. 220, 2019.

- Lan Chen, Lin Gao, Jie Yang, Shibiao Xu, Juntao Ye, Xiaopeng Zhang, and Yu-Kun Lai, Deep deformation detail synthesis for thin shell models, arXiv 2021.

- Meng Zhang, Tuanfeng Wang, Duygu Ceylan, Niloy J. Mitra, Deep Detail Enhancement for Any Garment, Eurographics 2021.

**Author response:** Thanks for the good question.

**Author action:** We added the manuscript by in Section *Related Work*.

**Reviewer#2, Concern #2:** The authors mentioned the flickering and over-shrinkaged problems at first but they seems not solve all these problems but boundary oscillation. I would like to see more results solving these two problems.

**Author response:** We conducted a new experiment and updated the manuscript accordingly. (see Figure 13).

**Author action:** We added the manuscript by “Line 413 in Section *Results and Discussion*”.

**Reviewer#2, Concern #3:** One of the main contribution is the anisotropic constrained-boundary network to generate stable boundaries. However, this blur boundary problem is due to the convolutional operation at the image boundaries. It is not the special problem in geometry images but a common issue in image super-resolution. You can test the SOTA SR model. In image SR field, researches often calculate the PSNR value after cropping boundaries. Besides, in mesh generation, Chen et.al used padding operations to solve the boundary problems. I would like to see more convincing results of your contribution.

**Author response:** Thanks for the good question. We agree with you that the boundary problem occurs as a result of the convolutional operation on the image boundary. This problem is overcome by excluding the boundary part in the image processing, but we have developed a more reliable method because the boundary can exist in a variety of shapes in the geometry image of cloth. Because each pixel in a geometry image corresponds to a single vertex position, we tried to solve this problem using a method other than image SR. Chen et al. proposed a technique to perform SR by converting cloth into geometry image and eased the problem that appears near the boundary by padding. This boundary issue is not significant in image SR, but it becomes critical when restoring from a 2D geometry image to a 3D geometry. As Chen et al. mentioned, padding is not a perfect solution to this problem; it is an empirical value determined for each scene, and when significant deformation occurs, the boundary problem worsens. We conducted a new experiment on this issue and updated the manuscript accordingly (see Figure 13).

**Author action:** Thanks for the nice advices. We added the manuscript by “Line 413 in Section *Results and Discussion*”.

**Reviewer#2, Concern #4:** You mentioned that "It does not work properly ... but only properly in SRResNet ...", but your method is also a residual superresolution network. Is there any difference? And I think this network is not the boundary solution.

**Author response:** As you mentioned, our method is a kind of residual SR network. However, our method is distinct from the original SRResNet since the features to learn in each ConvNet are different, as are the forms of ConvNets that comprise the network (GeometryNet, BoundaryNet, EnhanceNet). However, we believe that the use of the residual network when learning these subnetworks is similar to SRRestNet.

**Author action:** Thanks for the good question.

**Reviewer#2, Concern #5:** The paper only use square and rectangular meshes as examples. Please give more results to demonstrate the correctness of your boundary network.

**Author response:** We conducted experiments on rectangular meshes to check the boundary problem's sensitivity in an ideal environment. This experiment can also be extended to work with more complicated meshes. The method applied to non-rectangular shapes can also be done in previous studies, which we added to the manuscript: Prior to sampling, a parametrization step is required to convert non-rectangular meshes to rectangular structures. There are several techniques to parametrization, including the widely used As-Rigid-As-Possible (ARAP) method.

**Author action:** Thanks for the good question. We added the manuscript by “Line 432 in Section *Results and Discussion*”.

**Reviewer#2, Concern #6:** The writing and organization in this paper needs to be improved. The authors use long sentences and the logic between sentences needs to be improved.

For example:

- Page1, "an anisotropic ... networks ... technique" -> "an anisotropic ... network" .

- Page1, "As a training set to be provided as an input to the neural network" too long

- Page1 "details including wrinkles" repeated.

- Fig 2. Which ANN did you use ? Please give citations.

- spelling issues line 246 "ground-true" -> "ground-truth"

**Author response:** We agree with your opinion.

**Author action:** Thanks for the nice advices.
