# Supplementary material for: Geometry image super-resolution with AnisoCBConvNet architecture for efficient cloth modeling
Source: PLoS One. 2022 Aug 24;17(8):e0272433. doi: 10.1371/journal.pone.0272433 (PMC9401171; doi:10.1371/journal.pone.0272433)
Supplement: Attachment — Submitted filename: Response_to_Reviewers.docx [file pone.0272433.s003.docx]

**Reviewer#3, Concern #1:** The writing in this paper needs to be improved. For example: Page6, "To alleviate the problems of ... when reconstructed from ... to ... " lack of sentence components

**Author response:** Thanks for the good question. The sentence your pointed out has been corrected to be clearer and more detailed.

**Author action:** We modified the manuscript.
